# Delineating season-specific hotspots: A spatio-temporal risk assessment of neck blast disease in rainfed and irrigated ecosystems of Cumilla District, Bangladesh

**DOI:** 10.1371/journal.pone.0358537

**Published:** 2026-09-21

**Authors:** Md. Mamunur Rashid, Niaz Md. Farhat Rahman, Nazmul Haque, Mohammad Ashik Iqbal Khan, Mohammad Salim Mian, Mohammod Hossain, Md. Rejwan Bhuiyan, Abul Bashar Md. Zahid Hossain, Abul Bashar Md. Anwar Uddin, Quazi Shireen Akhter Jahan, Md. Rafiqul Islam

**Affiliations:** 1 Plant Pathology Division, Bangladesh Rice Research Institute, Regional Station, Cumilla, Bangladesh; 2 Agromet Lab, Bangladesh Rice Research Institute, Gazipur, Bangladesh; 3 Department of Statistics, Shahjalal University of Science & Technology, Sylhet, Bangladesh; 4 Plant Pathology Division, Bangladesh Rice Research Institute, Gazipur, Bangladesh; Canakkale Onsekiz Mart University, TÜRKIYE

## Abstract

Rice blast disease, caused by *Magnaporthe oryzae*, is a major and foremost constraint to rice production in Bangladesh which threaten the food security and farmer livelihoods. This study delineates season-specific hotspots of the devastating rice Neck Blast Disease (NBD) across rainfed (Aman) and irrigated (Boro) ecosystems in the Bangladesh’s most intensive rice cultivation area Cumilla District using spatio-temporal risk assessment. Multi-year field data (2017–2025) were analyzed through hierarchical clustering, Moran’s I, Local Indicators of Spatial Association (LISA), and geostatistical interpolation techniques, including ordinary and indicator kriging. Results revealed spatial heterogeneity and inter-annual variability in disease severity. Nangalkot emerged as a persistent Aman-season hotspot, with mean NBD severity reaching 45–60%, while peak Boro-season severity reached 60–75% in several Upazilas, including Cumilla Sadar Dakshin, Debidwar, Barura, Burichang, and Nangalkot. Climatic trends correlated with neck blast disease intensification, particularly during reproductive stages. Indicator kriging effectively identified high-probability risk zones (>20% severity), enabling precise mapping of localized outbreaks. Findings of this study underscore the need for location-specific, adaptive management strategies integrating resistant cultivars and predictive modeling. This research offers actionable insights for policymakers and extension services to mitigate blast epidemics and safeguard Bangladesh’s rice production under evolving climatic conditions.

## Introduction

Agriculture, especially rice (*Oryza sativa* L.) production, is central to Bangladesh’s economy and food security [1]. Despite challenges from population growth, limited arable land, and frequent natural disasters, Bangladesh has achieved self-sufficiency in rice through advances in both crop genetics and management [2, 3]. As the staple food for over 170 million people, rice production accounts for approximately 46% of the crop sector’s gross domestic product (GDP) and provides the bulk of caloric (70%) and protein intake (56%) for the population [4, 5]. In Bangladesh, rice production mainly concentrated across three main seasons: Aus, Aman (rainfed) and Boro (irrigated) where Aman and Boro crops contribute the vast part of its total production. Given the projected population increase and the concurrent challenges posed by climate variability—driven by both internal variability and external climate forcings in the region [6–15]—maintaining and enhancing the current yield of approximately 39 million tons remains paramount [4,16–18].

However, despite the significant advances in adopting high-yielding varieties and irrigation technologies, disease of rice particularly blast disease poses the most devastating threat on production of rice [19]. The disease manifests in various stages, but infection at the panicle neck which known as Neck Blast (NBD) is particularly consequential as it directly interferes with grain filling [20, 21]. This leads to chalky kernels or complete sterility and causing immediate, catastrophic yield losses. Studies have estimated that under epidemic conditions, losses attributable to blast can range from 60% up to 100% in severely affected fields, posing a direct threat to farmer livelihoods and national stability [22].

Crucially, the confluence of high humidity, increase in temperatures, and intense monoculture during the rice plant’s vulnerable reproductive stage creates a highly congenial environment for severe Neck Blast development during the critical panicle initiation and flowering stages in Bangladesh [23–26]. Previous studies have reported that Neck Blast development is favored by moderate temperatures of approximately 25–28°C, high relative humidity above 93%, and prolonged leaf wetness of 7–14 hours, which enhance conidial germination, appressorium formation, and infection efficiency of *Magnaporthe oryzae* [27]. Multi-year weather analysis of the study region (Cumilla District) confirms an observable rising trend in maximum temperature across both the *T. Aman* and *Boro* seasons which indicates a climatic shift that provides a more favorable thermal environment for the pathogen [28]. While Neck Blast is known to affect both major growing seasons, disease incidence and severity are often high in the irrigated *Boro* season (November–May) and also demonstrate severe localized impact in the rainfed *T. Aman* season (July–December) [29,30].

The main objective of this study is addressing the critical knowledge gap concerning the spatial and temporal dynamics of Neck Blast disease under the distinct environmental pressures of the *Aman* and *Boro* seasons across the Cumilla District. This research seeks to: (1) quantify the annual incidence and severity of Neck Blast in major rice-growing regions and establish patterns of chronic risk; (2) identify and delineate significant spatial clustering of disease severity using Moran’s I hotspot analysis; and (3) utilize Indicator Kriging (IK) to delineate specific, high-probability risk zones (e.g., severity exceeding 20%) during the Boro season to aid in proactive disease management. The novelty of this study lies in its focus on season-specific NBD hotspots in Cumilla District, comparing rainfed Aman and irrigated Boro ecosystems using multi-year field data and geospatial techniques to identify persistent and shifting disease-risk zones. Ultimately, the findings will provide actionable, data-driven insights necessary for breeders, extension workers, and policymakers to develop targeted, sustainable management strategies, specifically through the deployment of stable, long-term resistant cultivars, to safeguard Bangladesh’s vital rice production base against this enduring and explosive phytopathological threat.

## Methodology

**Data:** The study was conducted by gathering data from 7 Upazilas (Debidwar, Burichang, Chandina, Cumilla Adarsha Sadar, Cumilla Sadar Dakshin, Barura, and Nangalkot) in Cumilla District for the Aman season during the year 2017–2024 and from 10 Upazilas (Muradnagar, Debidwar, Burichang, Chandina, Cumilla Adarsha Sadar, Cumilla Sadar Dakshin, Barura, Laksham, Chauddagram, and Nangalkot) in Cumilla District for the Boro season during the 2018−19–2024−25 season. Follow the link to the S1 File to see the study regions broken down by year. Disease data was recorded from available sampling sites in each selected Upazila during each study year and the number of sampling sites fluctuated among Upazila and years depending on the accessibility of the field, the occurrence of diseases and survey coverage. Not all the same fields were necessarily monitored every year, but it was in the form of repeated Upazila-level field surveys. Because field selection was partly constrained by accessibility and disease occurrence, some sampling bias may have been introduced, and the surveyed fields may not fully represent all rice fields within each Upazila.

The 0–9 scale based on the Standard Evaluation System (SES) for rice was used to compute disease grading [31]. Using formula (1), the severity of neck blast was converted to a Percent Disease Index (PDI), as shown below.


$$\begin{array}{c} PDI=\;\frac{Sum\;of\;Individual\;ratings}{No.\;of\;panicles\;assessed\;\times \;Maximum\;disease\;grade\;value}\;\times \text{1}\text{0}\text{0} \end{array}$$ (1)

### Estimation of yield loss

Yield loss associated with neck blast disease was estimated from neck blast incidence using the empirical relationship described by Hossain et al. [32], following Padmanabhan [33]. Predicted grain yield was calculated as:


$$\begin{array}{c} Y=\text{1}\text{9}\text{6}\text{7}.\text{9}\text{5}-\text{1}\text{8}.\text{7}\text{2}X \end{array}$$

where Y is the predicted grain yield (lb/ha) and X is neck blast incidence (%). Predicted yield was converted to (t/ha) using a conversion factor of 0.000453592. The disease-free yield predicted by the model (approximately 0.89 t/ha was used as the reference yield, and percentage yield loss was estimated as:


$$\begin{array}{c} \text{Yield loss }(\%)=\frac{\text{0}.\text{8}\text{9}-Y_{\text{t}/\text{ha}}}{\text{0}.\text{8}\text{9}}\times \text{1}\text{0}\text{0} \end{array}$$

The resulting yield-loss estimates were summarized by Upazila and year for comparison of the spatial and temporal effects of neck blast disease.

**Cluster Analysis:** To determine distances between Upazilas, an aggregative hierarchical cluster analysis was performed using the average linkage approach depending on the severity of NBD. Average linkage was selected because it provides a balanced clustering approach by considering the average pairwise distance between observations in two clusters. This method is less sensitive to chaining effects than single linkage and less restrictive than complete linkage, making it suitable for grouping Upazilas with similar Neck Blast Disease severity patterns across years. Silhouette analysis was performed to validate the hierarchical clustering results and to identify the optimal number of clusters based on average silhouette width. Data optimization and cluster analysis were carried out using R’s ‘hclust’ function (version R-4.0.3). In an average linkage hierarchical clustering, the distance (L) between two clusters (r,p) is the distance between two points, as stated by formula (2):


$$\begin{array}{c} L(r,\;p)=\;\frac{\text{1}}{n_{r}n_{p}}\sum_{i=\text{1}}^{n_{r}}\sum_{j=\text{1}}^{n_{p}}D(X_{ri},X_{pj}) \end{array}$$ (2)

where X and Y represent the observations from clusters r and p, respectively.

**Geostatistical analysis:** To assess the spatial pattern of NBD, two major methods were used: surface interpolation and point pattern analysis. point-pattern-optimized hotspot analysis were utilized to establish cluster patterns for the occurrence of NBD. For creating spatial maps and determining possible risk zones, interpolation methods such as inverse distance weighting (IDW), ordinary kriging (OK) and indicator kriging (IK) were used. All spatial maps were prepared using the WGS 84/ UTM Zone 46N projected coordinate system (EPSG:32646), and scale bars and north arrows were added where applicable.

**Point pattern analysis:** Spatial autocorrelation was examined with Moran’s I and LISA statistics, tuned from the closest sampling points. Point-pattern hotspot analysis was conducted in R using the sf, spdep, and gstat packages; distance-based spatial weights were generated from Upazila coordinates, and spatial clustering was assessed using Global Moran’s I and LISA statistics at the 5% significance level. LISA helped in detecting spatial agglomeration, and significance was detected from the respective p-values in Table 1. Moran’s I index of every spatial unit i was calculated using Eq. (3).

**Table 1:** Classification criteria of Location using Moran’I.

Local Moran’s I	P values	Mean Comparison	Classification
Positive	Significant	Above mean	High–High (HH) cluster
Positive	Significant	Below mean	Low–Low (LL) cluster
Negative	Significant	Above mean	High–Low (HL) outlier
Negative	Significant	Below mean	Low–High (LH) outlier
	Not significant	—	No cluster


$$\begin{array}{c} I_{i}=\;\frac{\left(x_{i}-\overset{―}{x}\right)}{m^{\text{2}}}\;\sum_{j}w_{ij}(x_{j}-\overset{―}{x}) \end{array}$$ (3)

where

x_i_ = value at location *i*

$\overset{―}{x}$ = mean of variable

$m^{\text{2}}=\;\frac{\sum_{k}{\left(x_{k}-\overset{―}{x}\;\right)}^{\text{2}}}{n}$ (Variance)

w_ij_ = weight matrix

The spatial weight matrix (w_ij_)for Moran’s I and LISA analysis was constructed using a distance-based approach, where neighboring relationships among Upazilas were defined from their geographic coordinate. Weights were assigned by proximity, so that Upazilas that are close to each other are spatial neighbors.

Classifying each location using Moran ‘I, P-value and mean:

### Spatial interpolation

Using the spatial interpolation approach, the values in the unsampled locations were predicted; for example, the NBD severities at sites (X1, X2…. Xn) are (Z1, Z2…. Zn). Spatial interpolation can be used to estimate the Z values at new point X. Using IDW and OK methods, the diseased surface area was calculated. An unsampled site’s IDW can be written using the formula (4):


$$\begin{array}{c} F(i)=\;\sum_{i=\text{1}}^{m}W_{i}Z\left(r_{i}\right)=\;\frac{\sum_{i=\text{1}}^{m}\frac{Z(r_{i})}{{\left|r-r_{i}\right|}^{p}}}{\sum_{j=\text{1}}^{m}\frac{\text{1}}{{\left|r-r_{j}\right|}^{p}}} \end{array}$$ (4)

Where, m represents the number of neighboring points and p indicates the parameter.

From the omitted point, using leaving one-out-cross validation the interpolated values are compared with the actual values.

The spatial correlation of the random function Z(X0) can be estimated using the interpolation method known as Kriging. Using the formula, the expected values of variable Z at unsampled point X0 are determined by formula (5).


$$\begin{array}{c} \gamma (d)=\;\frac{\text{1}}{\text{2}}\sum {\left\{\left[\hat{Z}\left(X_{\text{1}}\right)-Z\left(X_{\text{2}}\right)\right]\right\}}^{\text{2}} \end{array}$$ (5)

For OK technique, the following formula (6) has been used:


$$\begin{array}{c} \hat{Z}\left(X_{\text{0}}\right)=\;\sum_{i=\text{1}}^{n}\lambda_{i}\hat{Z}\left(X_{i}\right) \end{array}$$ (6)

where Z represents the variable of interest at spatial coordinates Xi and Xo. n is the number of neighbors connected with the sampling point; λ_i_ is the weight associated with sampling point Xi and the i^th^ observation point. Semivariograms are used to calculate the closest neighbor indexed based on average spatial variability and NBD severity. It is defined as follows in (7):


$$\begin{array}{c} \hat{y}(h)=\;\frac{\text{1}}{\text{2}N(h)}\;\sum_{i=\;\text{1}}^{N(h)}{\left[Z\left(X_{i}\right)-Z\left(X_{i}+h\right)\right]}^{\text{2}} \end{array}$$ (7)

where *γ(h)* is the semivariance for the interval distance class *h*, *N(h)* is the number of data pairs of a given lag interval distance and direction, *Z (x*_*i*_*)* is the measured sample value at point *i*, and *Z (x*_*i*_ + *h)* is the measured sample value at position *I* + *h*.

Semivariogram values are fitted with spherical, exponential, Gaussian models and Matérn (Mat) model:

such as:

Spherical model:


$$\begin{array}{c} \hat{y}(h)=\;C_{\text{0}}+C\;\left[\text{1}.\text{5}\frac{h}{a}-{\left(\frac{h}{a}\right)}^{\text{3}}\right],\;if\;\text{0}\leq h\leq a. \end{array}$$

Exponential model:


$$\begin{array}{c} \hat{y}(h)=\;C_{\text{0}}+C\;\left[\text{1}-\text{exp}\{-\frac{h}{a}\}\right],\;for\;h\;\geq \text{0} \end{array}$$

Gaussian model:


$$\begin{array}{c} \hat{y}(h)=\;C_{\text{0}}+C\;\left[\text{1}-\text{exp}\{-\frac{h^{\text{2}}}{a^{\text{2}}}\}\right],\;for\;h\;\geq \text{0} \end{array}$$

Matérn (Mat) model:


$$\begin{array}{c} \hat{y}(h)=\;C_{\text{0}}+C\;\left[\text{1}-\frac{\text{1}}{{\text{2}}^{v-\text{1}}\Gamma (v)}{\left(\frac{h}{a}\right)}^{v}K_{v}(\frac{h}{a})\right],\;for\;h\;\geq \text{0} \end{array}$$

Where, C_0_ = nugget

C = partial sill

a = range parameter (scale

ν>0 = smoothness parameter

Γ(ν) = Gamma function

K_ν_ = modified Bessel function of the second kind

The Spherical, Exponential, Gaussian and Matérn are the models that have been tested, as they are the most commonly used models that represent various types of spatial dependence in geostatistical analysis. The accuracy of estimated data across models and methodologies was examined using accuracy measures such as average standard error (ASE), mean square error (MSE), and root mean square error (RMSE). The best-fitted semivariogram model was selected using leave-one-out cross-validation by comparing MSE, RMSE, and ASE, while also considering the plausibility of estimated spatial parameters, including nugget, sill, and range. Indicator kriging (IK) was utilized to identify disease-vulnerable locations when the severity of NBD exceeded 20% per field. However, for the 2021–22 season, when overall disease severity was very low, a 5% threshold was applied to capture spatial variation under low-prevalence conditions and to distinguish relatively vulnerable locations. NBD severity threshold was used as a practical operational cutoff for indicator kriging to identify areas with meaningful disease pressure and potential management concern; however, this threshold was applied as a risk-mapping criterion rather than as a universal biological threshold. Based on this, probability risk maps were constructed using the best-fitted semivariogram model. A similar method was used to create a color-coded map for ordinary kriging, with the contour symbol representing the higher risk areas of NBD in Cumilla’s various rice ecosystems.

### Ethics statement

This study did not involve human participants, human data, human biological materials, or animal subjects. The research was conducted exclusively on Neck Blast Disease of rice using field-level observations collected from agricultural plots. Therefore, ethical approval from an institutional ethics committee was not applicable, and informed written or verbal consent was not required.

## Result

### Weather conditions

**Fig 1:**
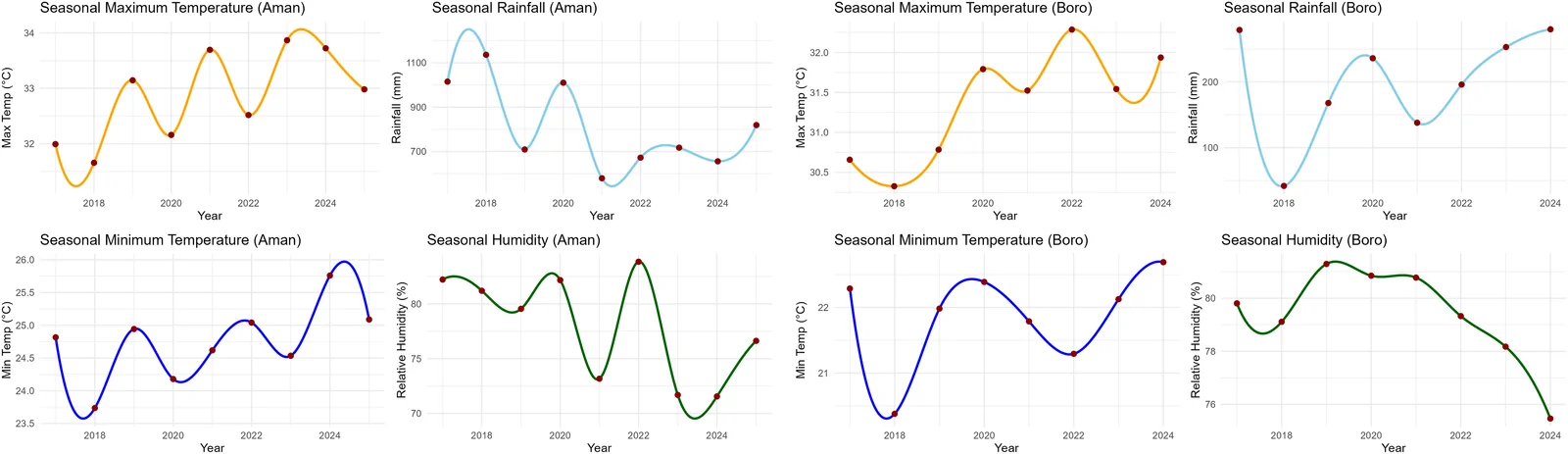
Trend of Climate parameter for both Aman and Boro Season.

During the Aman season in Fig 1, maximum temperature exhibited a clear rising trend between 2017 and 2024. Before 2020, it fluctuated between 32 °C and 33 °C, while after 2020 it ranged between 32 °C and 34 °C. A similar upward pattern was observed in the Boro season, where values varied between 29 °C and 33 °C. Precipitation, however, showed a declining trend in the Aman season. It peaked at around 1100 mm in 2018 but, after 2022, fluctuated within the range of 500–800 mm. In contrast, the Boro season displayed an increasing rainfall trend after 2018, reaching a peak of approximately 250 mm in 2020. Minimum temperature showed a slight upward trend in both seasons, ranging between 21 °C and 27 °C in Aman and 18 °C to 23 °C in Boro. Compared to Aman, relative humidity during Boro was lower (72–80%) and more variable, peaking between 2019 and 2020, followed by a steady decline thereafter. Mann–Kendall trend analysis showed that only maximum temperature during the Aman season had a statistically significant increasing trend (tau = 0.282, p = 0.041), while detailed results for all weather variables in both seasons are provided in S2 and S3 Files.

### NBD severity in different Upazila in Cumilla

**Fig 2:**
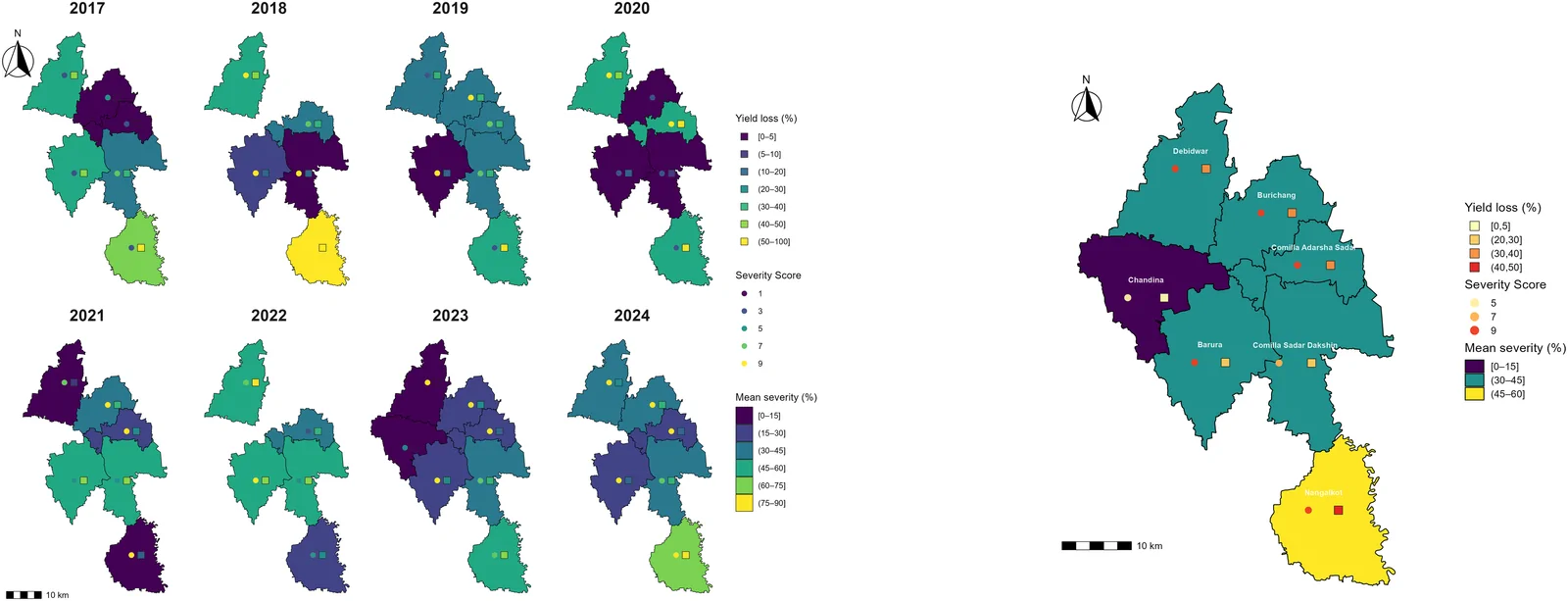
Neck Blast Disease severity map with Yield loss of Aman season between 2017 to 2024.

Fig 2 represent the descriptive visualizations of Neck Blast Disease (NBD) severity of Aman season that varies across the study area and year. Overall, highest mean severity is documented in Nangalkot, ranging from 45% to 60%, whereas Chandina shows the lowest mean severity (0–15%) with least severity across the year 2017–2024. Following that yield loss also highest at Nangalkot and Chandina shows the least based in this study.

In Debidwar, most of the time (2017, 2018, 2020, 2022) 45% to 60% mean severity is observed where in 2021 and 2023 it observed the least severity ranging from 0% to 15%. Debidwar experienced the highest yield loss (50–100%) in the year 2022 while other times it varied between 20% to 40%. In Burichang, the mean severity fluctuated mostly between 15% and 45%, with higher intensity during 2019, 2021 and 2024. Yield loss in Burichang generally ranged from 20% to 40% except for 2017 and 2020, when yield loss dipped to the lowest range (0–15%). Cumilla Sadar Dakshin exhibited moderate to high variation. In this region, mean severity was mainly between 15% and 60%. Following by mean severity most yield loss is also experienced this year. Barura displayed variable trends across the years, with mean severity mostly between 30% and 60%, and yield loss lying within the 30% to 50% range. Highest yield loss is observed in the year of 2017, 2021 and 2022.

Overall, the spatial pattern indicates that Nangalkot and Debidwar are the most vulnerable Upazilas for NBD in Aman season, while Chandina consistently remains the least affected both in terms of severity and yield loss.

**Fig 3:**
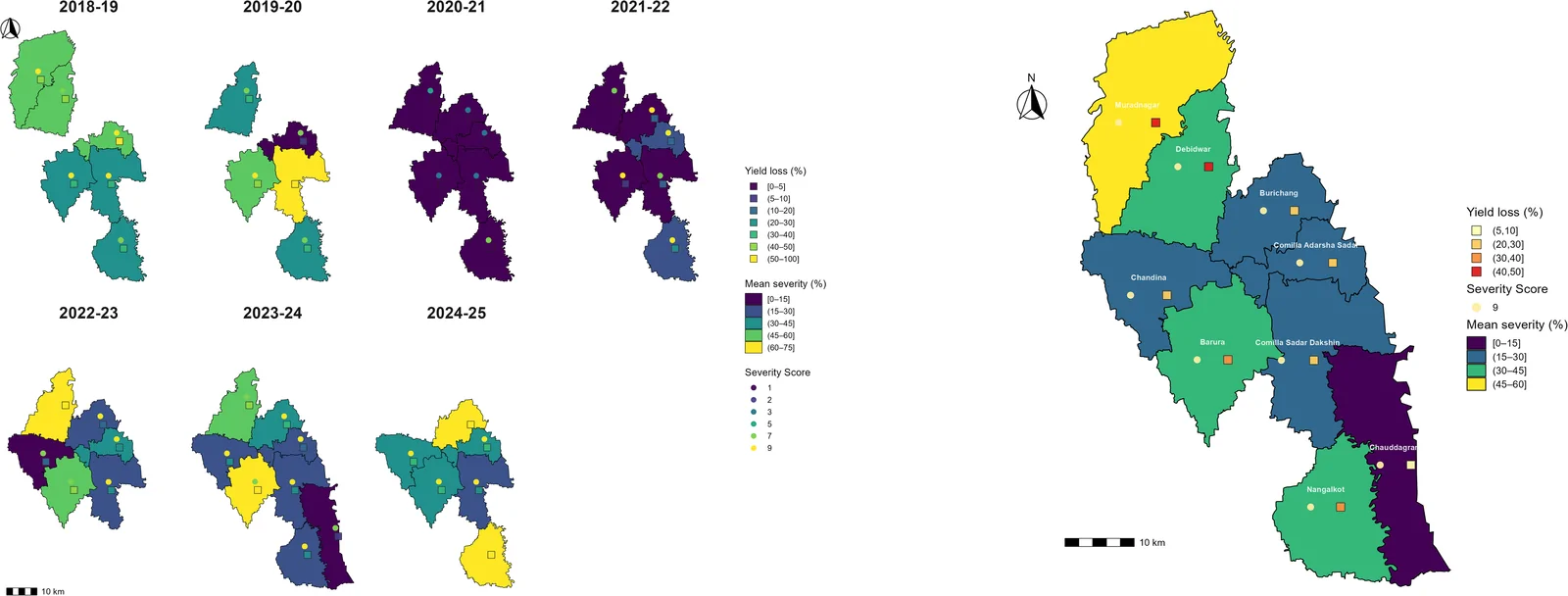
Neck Blast Disease severity map with Yield loss of Boro season between 2018 to 2025.

Fig 3 represents descriptive visualization of the mean severity and yield loss of Boro season in Cumilla Upazila across 2018–2025. Overall, it is evident that Muradnagar is more vulnerable and Chauddagram is considered as least for NBD in Boro season in terms of both mean severity and yield loss. It is notable that the 2020–21 and 2021–22 seasons showed the least severity during the Boro season compared to other years.

The highest severity, ranging from 60% to 75%, was observed in Cumilla Sadar Dakshin (2019–20), Debidwar (2022–23), Barura (2023–24), and both Burichang and Nangalkot (2024–25). With the exception of the 2020–2021 seasons, the least severity was recorded in Burichang (2019–20), Chandina (2022–23), and Chauddagram (2023–24). In terms of yield loss, the highest losses were reported in Burichang (2018–19), Cumilla Sadar Dakshin (2019–20), Debidwar (2022–23), Barura (2023–24), and again in both Burichang and Nangalkot (2024–25) that is highly associated with mean severity of disease.

### Hierarchical clustering

**Fig 4:**
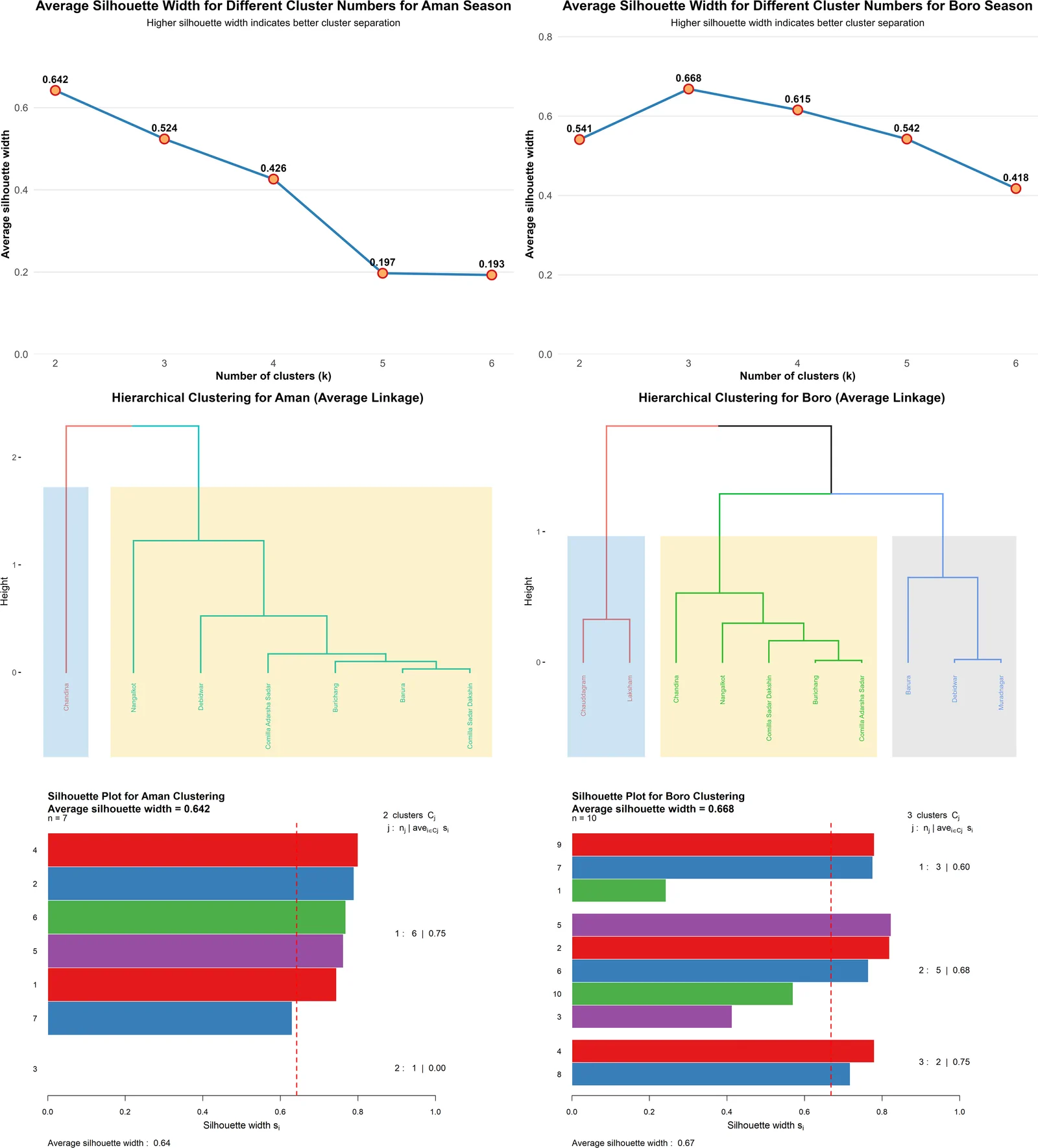
Hierarchical Clustering of Upazilas for (a) Aman and (b) Boro season (Average Linkage).

Hierarchical clustering using average linkage method is performed for two distinct seasons: Aman and Boro in Fig 4. The optimum number of clusters varied from season to season through cluster validation by silhouette analysis. For Aman season the two clusters (k = 2, average silhouette width = 0.642) showed the highest average silhouette width, which is a sign of a relatively good separation of the clusters. There was one cluster which consisted of Nangalkot, Debidwar, Cumilla Adarsha Sadar, Burichang, Barura and Cumilla Sadar Dakshin and another cluster for Chandina that has its own pattern of disease severity. For the Boro season, the maximum average silhouette width was found for three clusters k = 3, average silhouette width = 0.668, which also showed good separation between the clusters. The first cluster consisted of Chauddagram and Laksham; the second cluster comprised Chandina, Nangalkot, Cumilla Sadar Dakshin, Burichang, and Cumilla Adarsha Sadar; and the third cluster was Barura, Debidwar and Muradnagar during the Boro season. The results also suggest that the season-specific cluster structure is useful for identifying the Upazilas which have similar Neck Blast Disease severity patterns.

### Spatial point pattern analysis of NBD

Local Moran’s I and p-values were used to analyze the clusters and outliers. NBD cluster patterns, which indicate scattered and aggregated clusters of severity, were found at the district level during the Aman season in 2017 and 2024 and the Boro season in 2018–2025 (Supplementary S1–S4 Fig). The majority of the Upazilas were grouped together (at I > 0) based on positive I values, indicating positive spatial autocorrelation between them. Upazilas form another cluster when I value are negative. To find out about high risk or low risk region Lisa clustering method is used.

**Fig 5:**
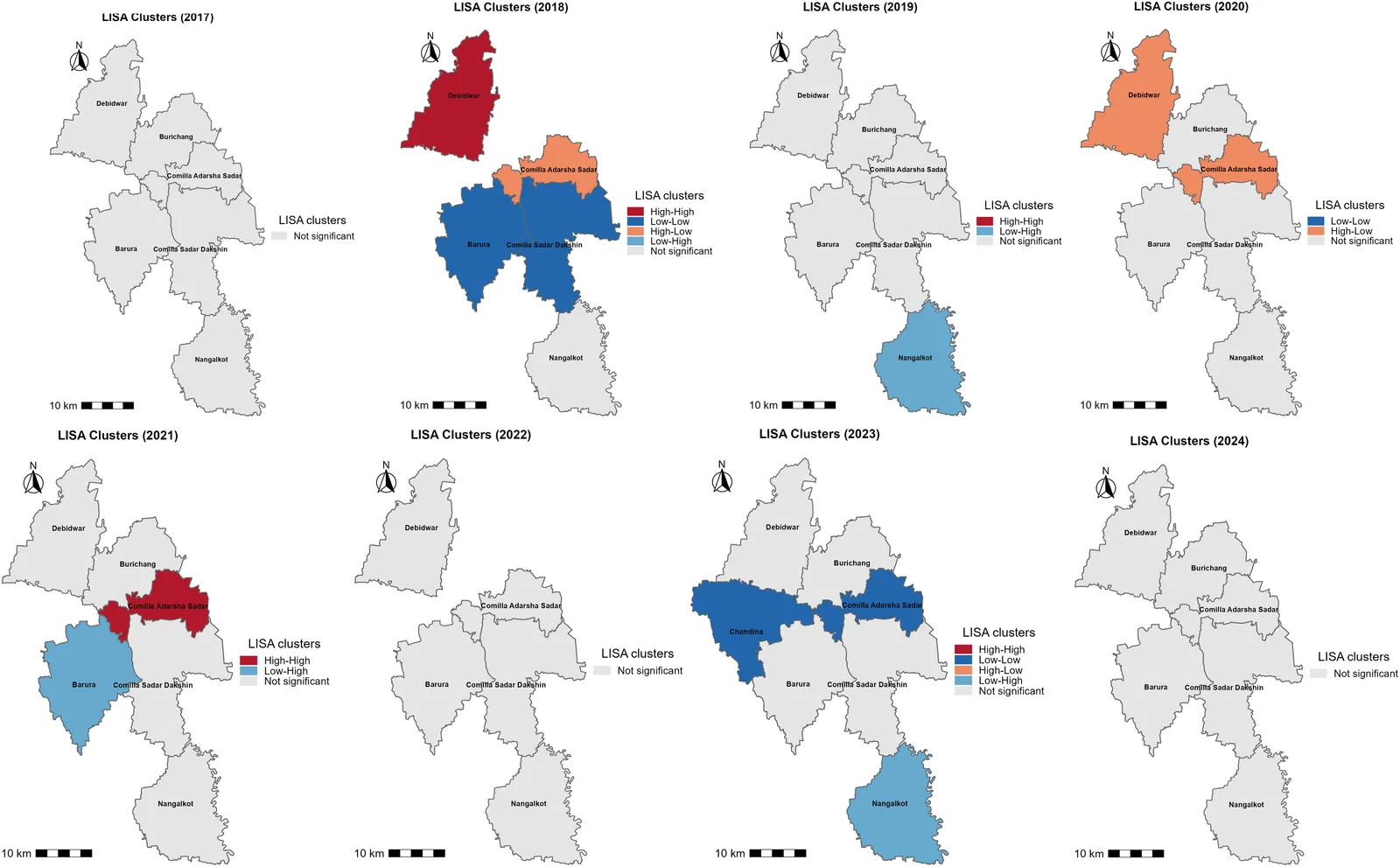
Spatiotemporal LISA Clustering of Neck Blast Disease Hotspots during Aman Season (2017–2024).

The LISA cluster maps in Fig 5 for the Aman season (2017–2024) show clear spatial heterogeneity in the severity of neck blast disease across Upazilas. Year-wise LISA analysis identified significant High–High clusters in Debidwar in 2018 and Cumilla Adarsha Sadar in 2021, indicating localized hotspots of high Neck Blast severity. Significant Low–Low clusters were observed in Chandina and Cumilla Sadar Dakshin in 2023, indicating localized areas of lower disease severity. Some years also show high–low or low–high outliers, indicating spatial contrast between neighboring Upazilas. These results lead to shifting hotspots and necessitate continued monitoring and location-specific interventions to manage blast risk effectively.

**Fig 6:**
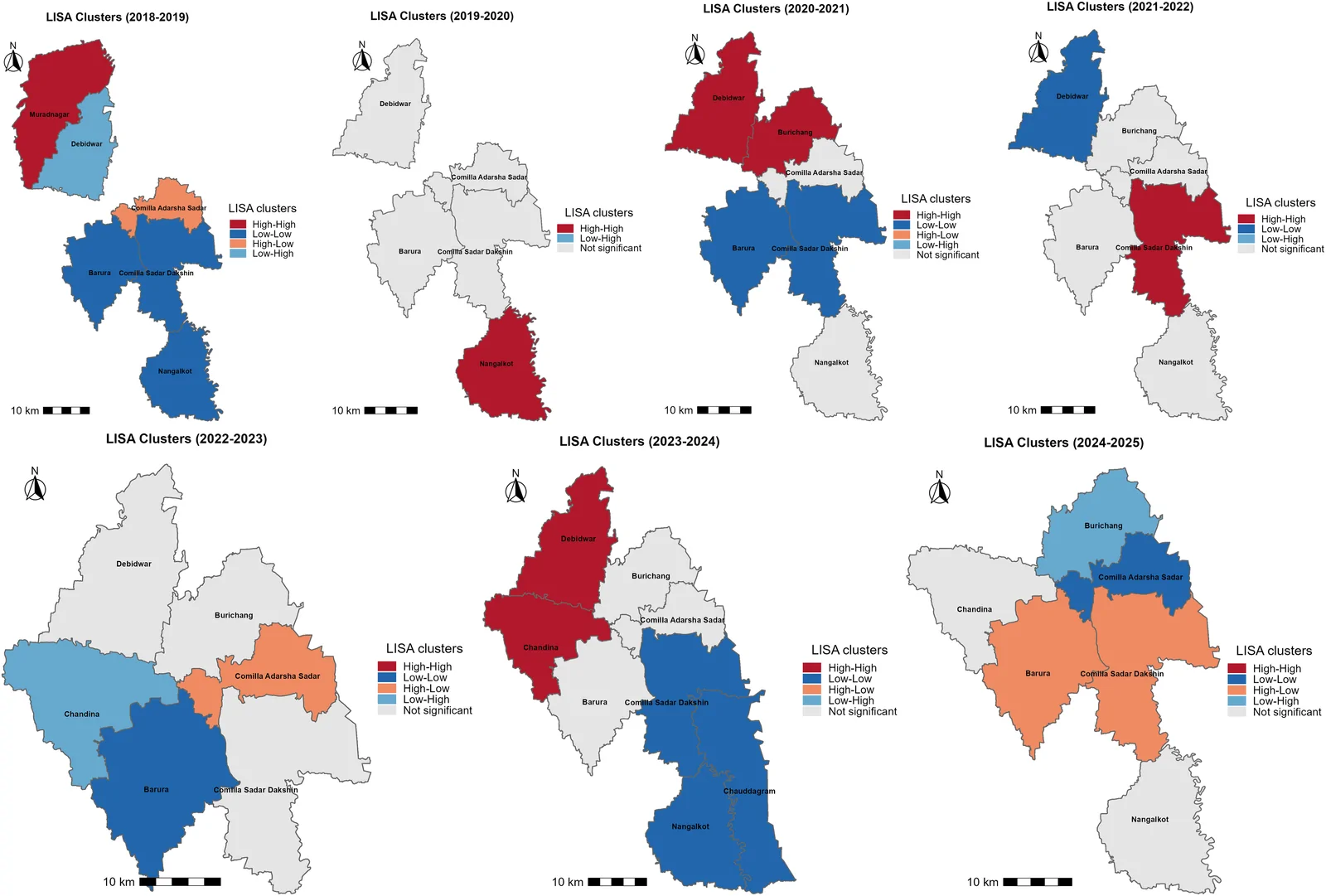
Spatiotemporal LISA Clustering of Neck Blast Disease Hotspots during Boro Season (2018–2025).

Fig 6 LISA cluster maps for Boro (2018–2025) illustrate dynamic spatial patterns of neck blast disease risk across Upazilas. High–High clusters are prevalent in Muradnagar (2018-2019), Nangalkot (2019-20), both Debidwar and Burichang (2020-21) and, Debidwar and Chandina (2023-24) marking them as stable hotspots that indicates the high disease severity surrounding by high severity, while Low–Low clusters feature in areas of lower disease severity in Barura, Cumilla Sadar Dakshin and Nangalkot (2018-19), Barura and Cumilla Sadar Dakshin (2020-2021), Barura (2022-23), Cumilla Sadar Dakshin, Chauddagram, Nangalkot (2023-24) and Cumilla Sadar Dakshin (2024-25). Transitional clusters (High–Low or Low–High) indicate spatial heterogeneity, showing shifting disease pressure between neighboring areas. These patterns call for location-specific monitoring and adaptive interventions to mitigate risks during the Boro season.

### Surface interpolation to the explicit spatial distribution

**Fig 7:**
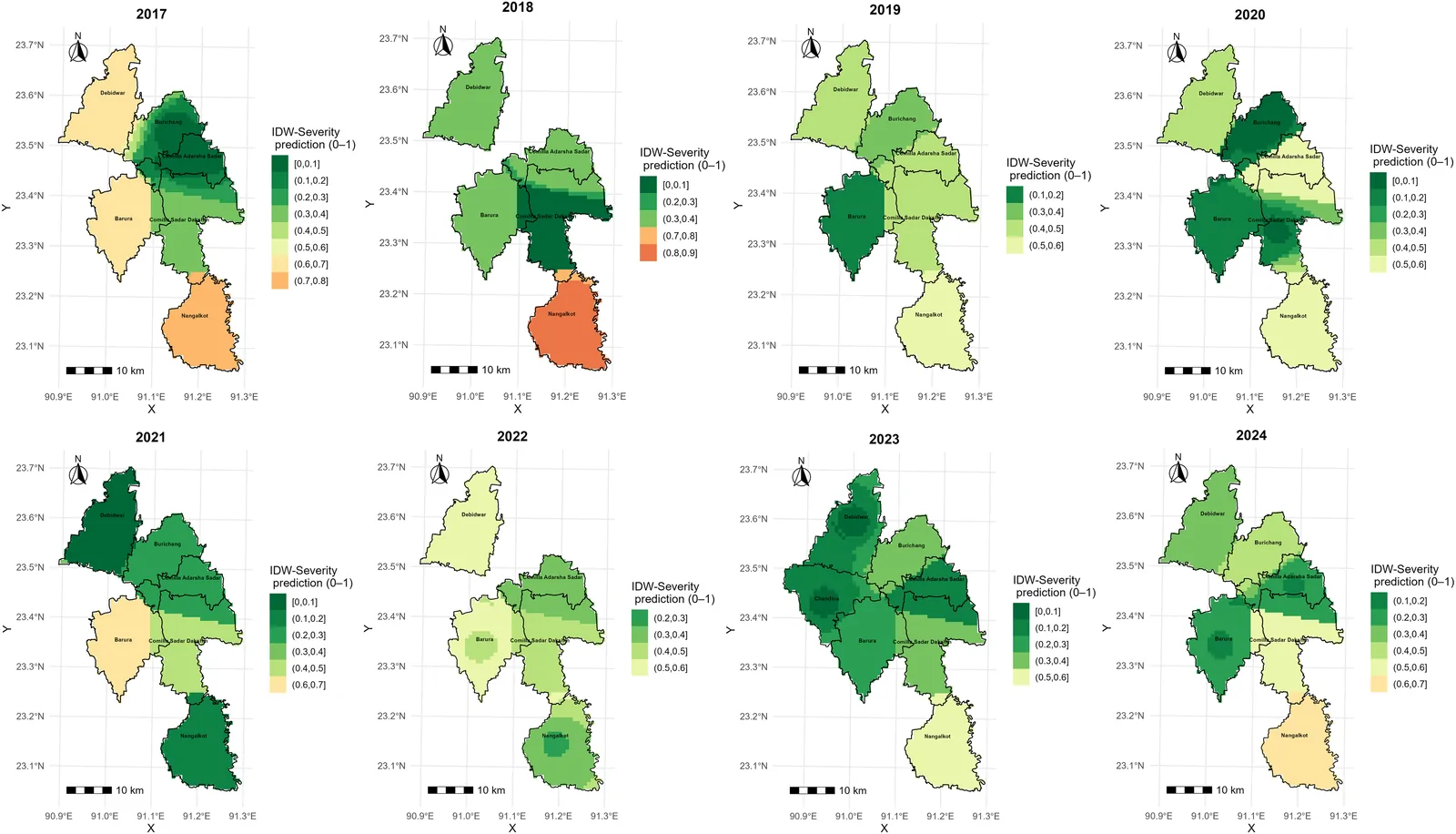
Interpolated disease severity maps of NBD for Aman Season were generated from 2017 and 2024 using the inverse distance weighted tool. Green to Red colors indicate lower to higher disease severity points in different Upazila of Cumilla. The maps were created using R software (version R-4.0.3).

**Fig 8:**
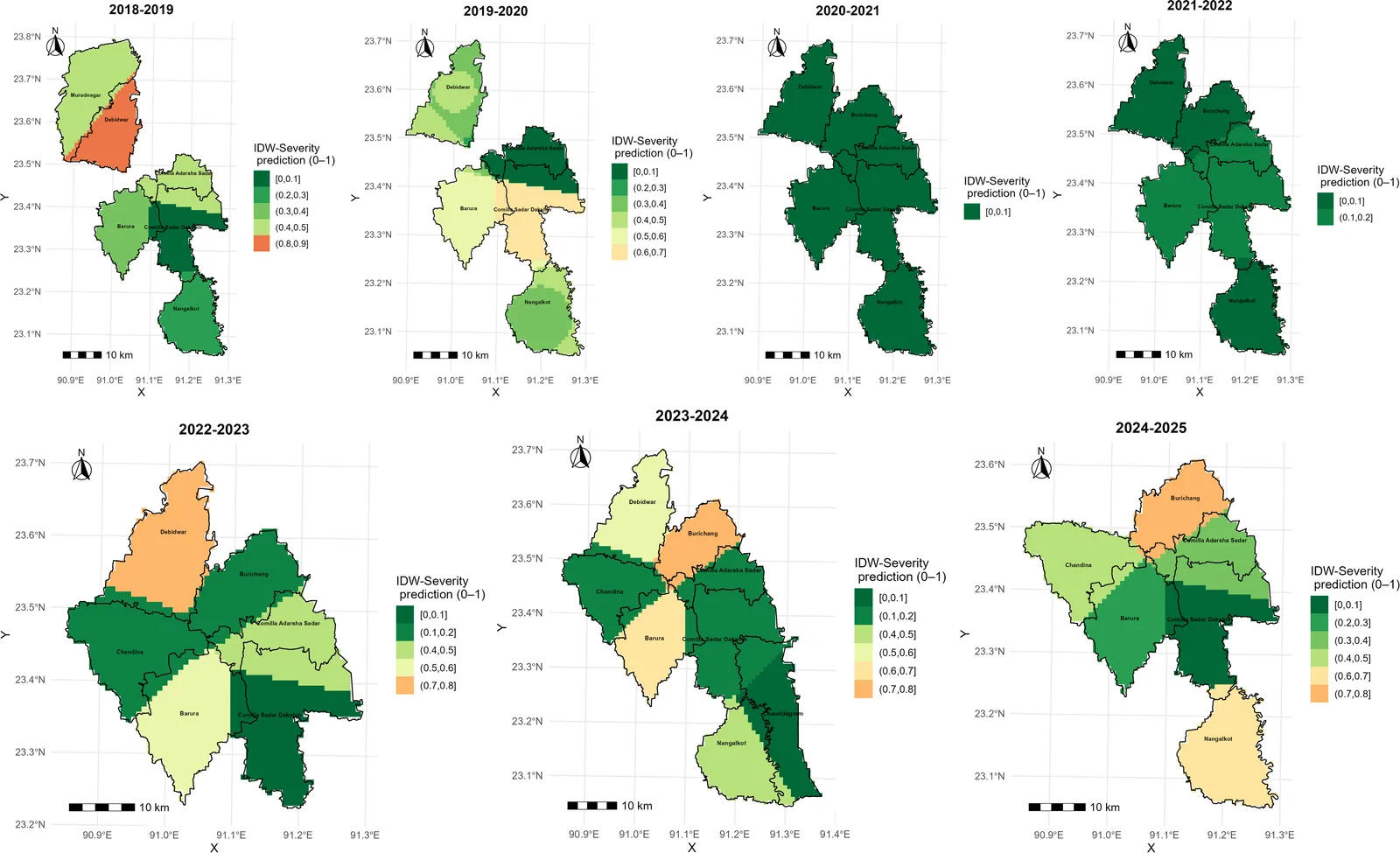
Interpolated disease severity maps of NBD for Boro Season were generated from 2018–19 to 2024–25 using the inverse distance weighted tool. Green to Red colors indicate lower to higher disease severity points in different Upazila of Cumilla. The maps were created using R software (version R-4.0.3).

Inverse distance weighting (IDW) interpolation method is used to identify missing cell values using weighted combinations of a set of sample points. Using this method, contour map of NBD for two distinct seasons, Aman and Boro, for several Upazila of Cumilla has been shown in Fig 7 and Fig 8.

Fig 7 shows that, Nangalkot Upazila is the main points of the Cumilla Upazila, which have greater disease proportions (ranging 70% to 90%) and became a risk prone area for NBD. According to this study, the middle portion of the study region is thought to be less disease-prone, with disease severity ranging from 20% to 30%. However, more concentrations should be made in 2017 (for Debidwar and Barura), 2021 (Barura), and 2024 (Cumilla Sadar Dakshin), where roughly 50% to 60% disease risk is observed.

Fig 8 shows that disease severity risk was lowest during the Boro seasons of 2020–21 and 2021–22. In contrast, the highest risk levels were observed in Debidwar (2018–19 and 2022–23), Burichang (2023–24 and 2024–25), Cumilla Sadar Dakshin (2019–20), and Barura (2023–24).

### Ordinary and indicator kriging

For ordinary and indicator kriging Spherical, exponential, and Gaussian semivariogram experimental models were used to determine the spatial patterns of NBD severity measurements. Based on cross-validation of the semivariogram results for Aman season (Table 1), which showed lower mean square error (MSE), root mean square standard error (RMSE), and average standard error (ASE) values.

From the above Table 2 it is seen that Overall, the Spherical model performed best in 2017, 2018, 2020, 2021, and 2024, showing the lowest RMSE values. In contrast, the Exponential model was selected for 2019, 2022, and 2023. Although the Gaussian model produced the lowest cross-validation error in 2023, its extremely large range and partial sill indicated an unstable and implausible fit; therefore, the Exponential model was preferred based on both predictive performance and parameter plausibility.

**Table 2:** Cross-validation results of semivariogram experimental models on NBD severity in Aman Season from 2017 to 2024 for *MSE* (mean square error), *RMSE* (root mean square standard error), *ASE* (average standard error).

Model	Range (km)	Partial sill	Nugget	MSE	RMSE	ASE
2017						
Spherical	19.9811	389.0023	260.0791	559.1432	23.6462	27.2315
Exponential	166.6619	1,647.0354	332.6231	600.0273	24.4955	25.1191
Gaussian	3,893.1402	13,318,870.9264	547.6348	662.1770	25.7328	30.2061
Mat	166.6623	1,647.0393	332.6233	600.0273	24.4955	25.1191
2018						
Spherical	17.5289	454.7926	303.1951	889.6730	29.8274	30.6045
Exponential	49.6252	559.6806	427.2508	990.0819	31.4656	28.3093
Gaussian	679.4885	1,324,097.5581	2,272.0321	1,953.8310	44.2022	64.1589
Mat	49.6252	559.6805	427.2510	990.0819	31.4656	28.3093
2019						
Spherical	17.5290	121.4978	80.9985	249.6530	15.8004	15.3574
Exponential	3.2940	1,895.8051	0.0000	245.3175	15.6626	47.4809
Gaussian	9.0211	234.2498	0.0000	257.6824	16.0525	16.0226
Mat	3.2940	1,895.8061	0.0000	245.3175	15.6626	47.4809
2020						
Spherical	15.6455	371.5367	251.0709	810.9678	28.4775	27.0981
Exponential	53.8602	453.8896	364.3021	928.2870	30.4678	24.8055
Gaussian	150.0331	1,417.9646	515.3487	855.1624	29.2432	25.7955
Mat	53.8602	453.8895	364.3022	928.2869	30.4678	24.8055
2021						
Spherical	34.6234	681.0578	0.0000	371.8725	19.2840	22.8343
Exponential	12.5779	1,016.2503	0.0000	374.7384	19.3582	30.1483
Gaussian	12.8748	570.1926	0.0000	382.6938	19.5626	21.9777
Mat	12.5778	1,016.2563	0.0000	374.7379	19.3581	30.1484
2022						
Spherical	19.5896	66.1887	43.8125	141.4908	11.8950	11.6057
Exponential	3.0427	2,729.6192	0.0000	140.1429	11.8382	58.3240
Gaussian	9.4404	144.7736	0.0000	143.4187	11.9758	13.1826
Mat	3.0426	2,729.6293	0.0000	140.1429	11.8382	58.3241
2023						
Spherical	20.5578	182.2374	107.2538	354.6472	18.8321	17.8904
Exponential	67.6913	319.6759	148.3786	240.2390	15.4996	16.4259
Gaussian	3,975.3337	13,570,528.0423	339.8002	85.9940	9.2733	23.6776
Mat	67.6913	319.6760	148.3787	240.2390	15.4996	16.4259
2024						
Spherical	39.7419	507.5758	0.0000	466.6841	21.6029	18.6135
Exponential	21.1292	700.4576	0.0000	484.1407	22.0032	21.5930
Gaussian	19.9515	396.0987	113.4933	492.4715	22.1917	19.1036
Mat	21.1292	700.4576	0.0000	484.1407	22.0032	21.5930

From Table 3, spherical model can be selected as optimal model for the season of 2018−19, 2019−20, 2022−23 and exponential outperform than others in 2021−22, 2023−24 and 2024−25 where gaussian model is perform best in 2020−21 based on minimum MSE values.

**Table 3:** Cross-validation results of semivariogram experimental models on NBD severity in Boro Season from 2018–19 to 2024–25 for *MSE* (mean square error), *RMSE* (root mean square standard error), *ASE* (average standard error).

Model	Range (km)	Partial sill	Nugget	MSE	RMSE	ASE
2018−19						
Spherical	15.6965	150.6535	0.0000	88.3476	9.3993	13.2841
Exponential	2.3733	1,550.7750	0.0000	93.3962	9.6642	43.1061
Gaussian	6.6533	131.5956	0.0000	90.9166	9.5350	12.4928
Mat	2.3733	1,550.7767	0.0000	93.3962	9.6642	43.1062
2019−20						
Spherical	17.4130	292.5107	195.0921	597.2154	24.4380	24.5521
Exponential	35.8263	260.3111	285.1725	650.2588	25.5002	22.6817
Gaussian	54.7903	185.4268	353.9159	633.9083	25.1775	22.1196
Mat	35.8263	260.3110	285.1726	650.2588	25.5002	22.6817
2020−21						
Spherical	17.5290	0.5133	0.3422	1.0408	1.0202	0.9982
Exponential	37.3340	2.1017	0.0000	0.5968	0.7725	0.9571
Gaussian	30.8227	1.0044	0.3541	0.5707	0.7554	0.8823
Mat	37.3340	2.1017	0.0000	0.5968	0.7725	0.9571
2021−22						
Spherical	20.0413	33.4553	22.9046	69.2396	8.3210	8.0249
Exponential	1.9064	9,725.9629	0.0000	68.5026	8.2766	107.9935
Gaussian	6.8659	108.6535	0.0000	69.4933	8.3363	11.2863
Mat	1.9064	9,726.0190	0.0000	68.5026	8.2766	107.9938
2022−23						
Spherical	11.0283	242.3127	162.1383	485.3447	22.0305	22.0124
Exponential	2.8623	2,108.8975	0.0000	493.0153	22.2039	50.1557
Gaussian	5.3316	591.7454	0.0000	487.3454	22.0759	26.5862
Mat	2.8623	2,108.8947	0.0000	493.0153	22.2039	50.1557
2023−24						
Spherical	10.8444	187.5803	122.6650	379.8289	19.4892	18.8228
Exponential	2.3915	2,196.8257	0.0000	379.4846	19.4804	50.0631
Gaussian	7.6473	149.9748	154.9372	384.6736	19.6131	18.5451
Mat	2.3915	2,196.8355	0.0000	379.4846	19.4804	50.0633
2024−25						
Spherical	16.5914	216.1858	147.5241	442.0996	21.0262	20.6357
Exponential	3.3861	2,555.5810	0.0000	433.4709	20.8200	55.0849
Gaussian	8.7480	369.3460	0.0000	449.7380	21.2070	20.1854
Mat	3.3861	2,555.5821	0.0000	433.4709	20.8200	55.0849

**Fig 9:**
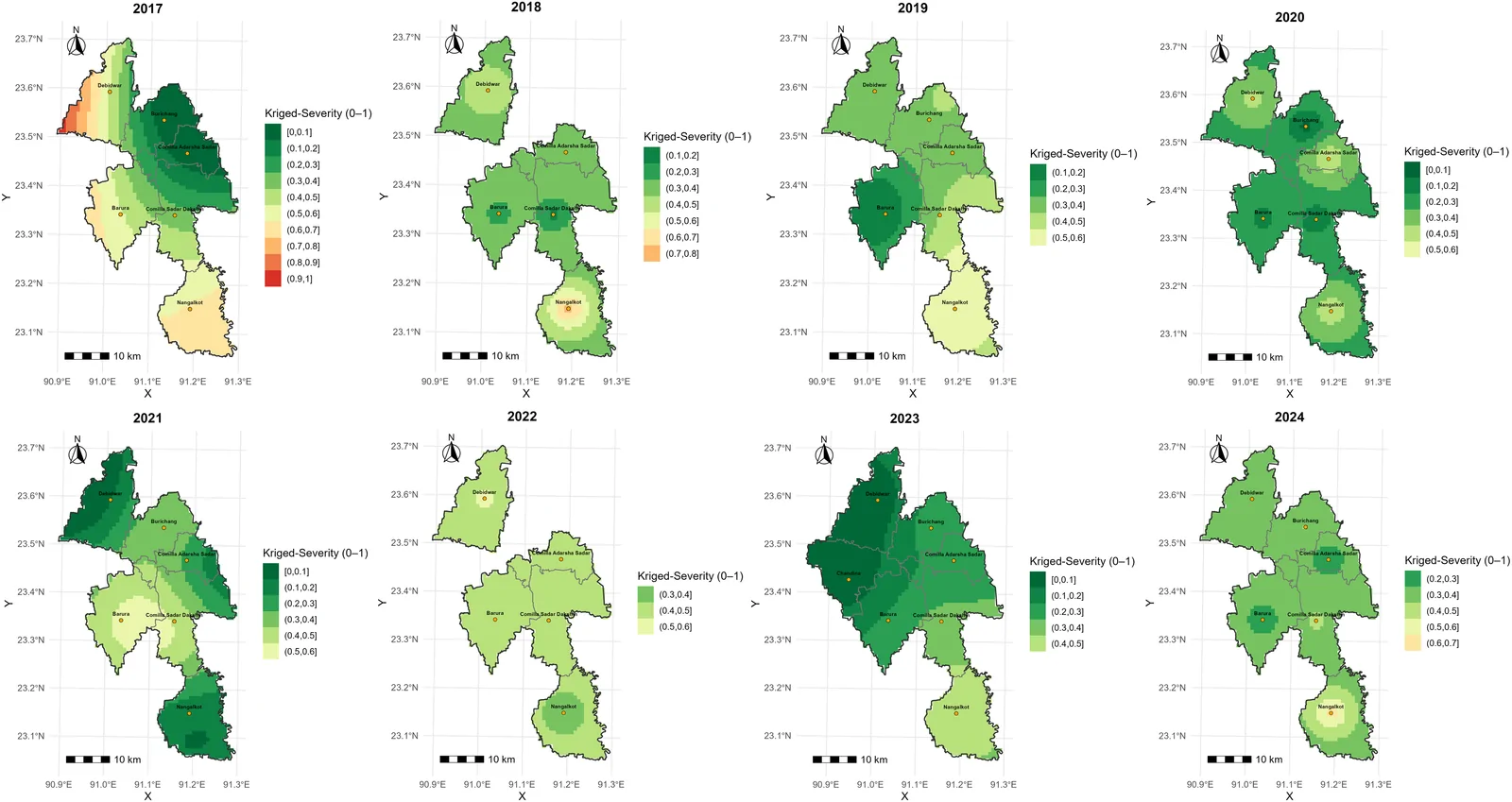
Ordinary kriging interpolated maps representing the spatial distribution of NBD in Aman season of Cumilla district during 2017 and 2024. Green to red-color coded surfaces depicts lower to higher disease severe points. The maps were created using R software (version R-4.0.3).

Using ordinary kriging interpolation in Fig 9, it is observed that in Aman season Nangalkot is more vulnerable, almost greater than 70% risk, to NBD across all study year. The district’s northeast and southeast regions (Debidwar, Barura, and Nangalkot) were more risky (>80%) in 2017 than others. The severity of the disease decreases between 2018 and 2020, but in 2021, Barura and Cumilla Sadar Dakshin demonstrate a risk of over 60% for NBD, which is prevalent in the majority of the study region in 2022. While Nagalkot continues to be a risk-prone area, the northern section of the district has relatively low disease severity in 2023. In the last year, the Upazila exhibits a fairly consistent moderate severity; nevertheless, Nangalkot exhibits a risk of over 70% for NBD, indicating that this is a high-risk location for NBD during this season.

**Fig 10:**
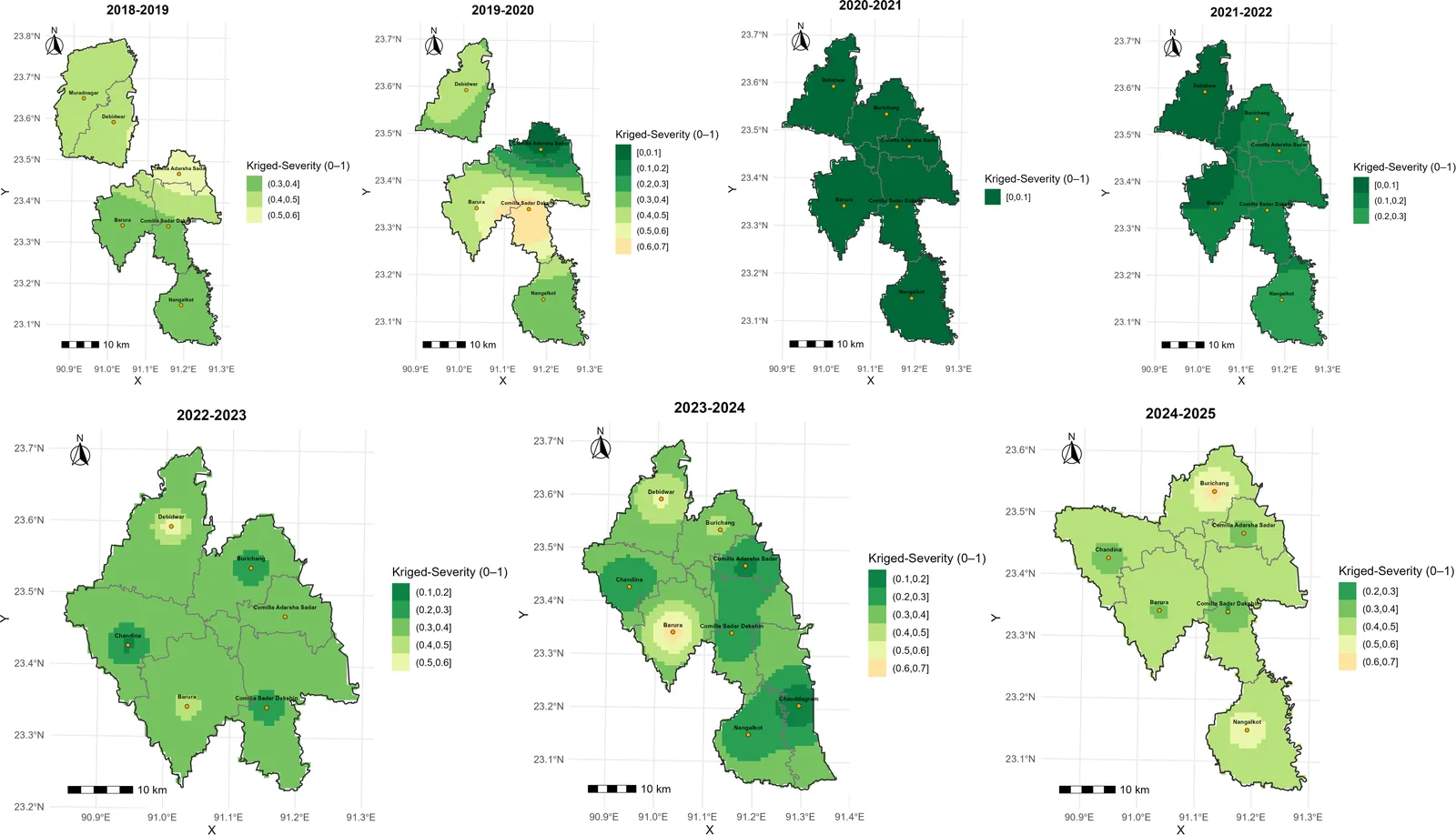
Ordinary kriging interpolated maps representing the spatial distribution of NBD in Boro season of Cumilla district during 2018 and 2024. Green to red-color coded surfaces depicts lower to higher disease severe points. The maps were created using R software (version R-4.0.3).

Fig 10 shows that the risk of NBD was significant prior to the 2021–22 Boro season. In 2018–19, the northern region had a disease severity of between 40% and 60% higher than the southern region. Cumilla Adarsha Sadar was the most dangerous of these, with a mean severity of about 60%. Cumilla Sadar Dakshin, which is in the center of the district, was identified as the NBD high-risk region the following year. Season 2021–2022 had the lowest danger by comparison. Risk has risen since this season. The majority of Cumilla District’s Upazilas can be regarded as high-risk areas in recent years. During this season, the risk of NBD is greater than 70% in Nangalkot and Burichang.

**Fig 11:**
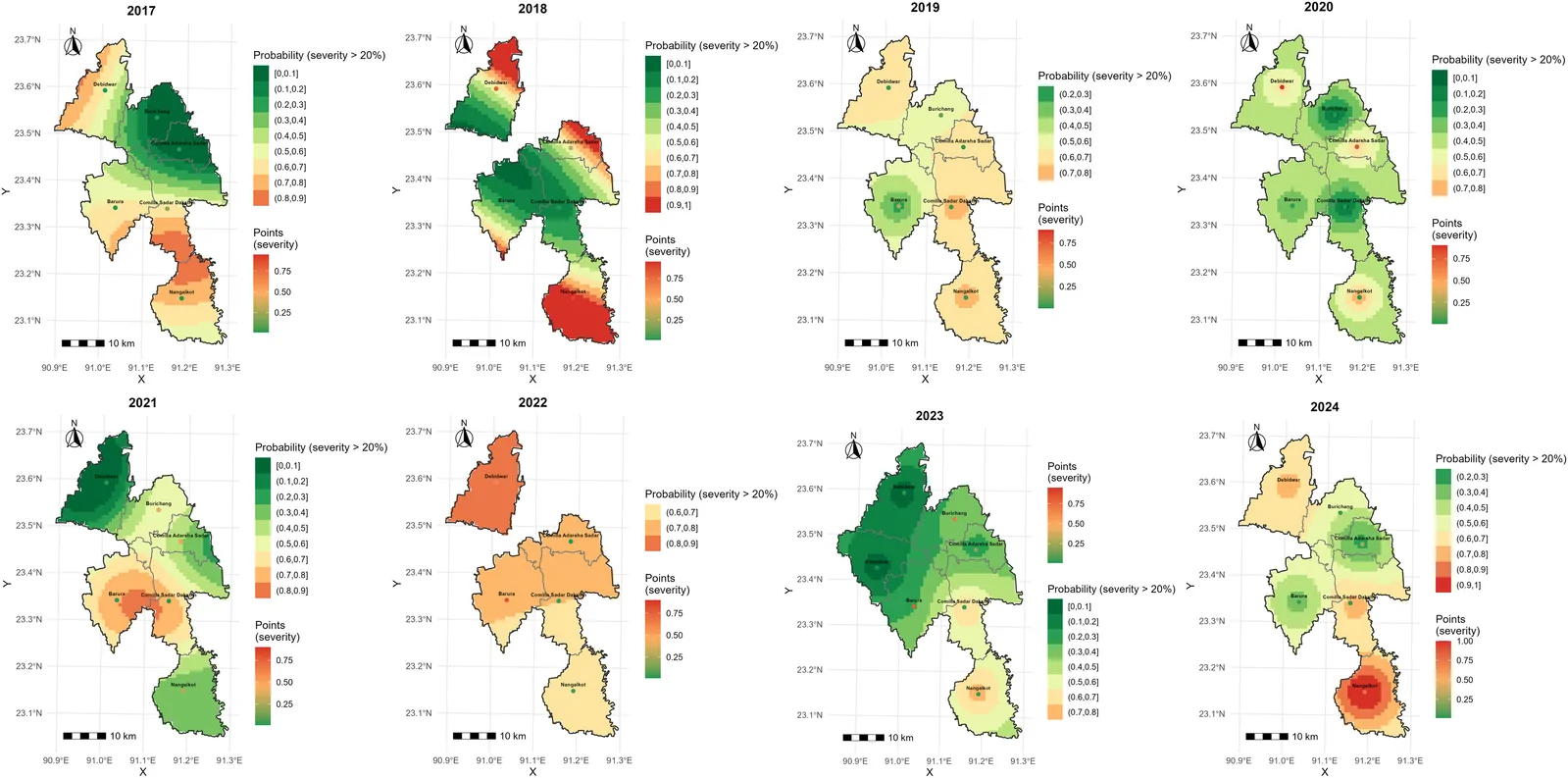
Neck blast disease probability distribution map for Cumilla in Aman season generated through semivariogram model information using indicator kriging. Green to red-colored points depict lower to higher levels of risk prone areas of NBD. The maps were created using R software (version R-4.0.3).

Fig 11 uses the indicator kriging approach to examine the spatial probability of Neck Blast Disease severity above 20% across the study Upazilas during the Aman seasons from 2017 to 2024. It is clear from this graph that in 2017, northern part of Debidwar and Nangalkot, and southern part of Barura were more susceptible to NBD. Between 50% and 80% was the range. In 2018, severe NBD (>90%) affected the northern parts of Debidwar and Cumilla Sadar and the southern half of Nangalkot, while mild NBD (10%–20%) affected the rest of the district. All Upazilas had a 50% to 70% risk in 2019, with the exception of Barura, where a 20% risk was noted throughout this period. While other study regions had a 10%–20% chance of disease in 2020, central Debidwar, Cumilla Adarsha Sadar, and Nangalkot had 60%–80% disease. While other Upazilas had risk levels below 30% in 2021, Barura, Cumilla Sadar Dakshin, and Burichang faced 60% to 80% risk. The northern portion of the district was at extremely high risk (>90%) for neck blast disease in 2022, while all Upazilas were at high risk. In 2023, every Upazila has a lower risk (<20%) with the exception of Nangalkot and the central region of Cumilla Sadar Dakshin. The danger of NBD increased in 2024, and Debidwar, Cumilla Sadar Dakshin, and Nangalkot may be considered high-risk areas. Nangalkot showed >90% probability of exceeding the specified NBD severity threshold during the period.

**Fig 12:**
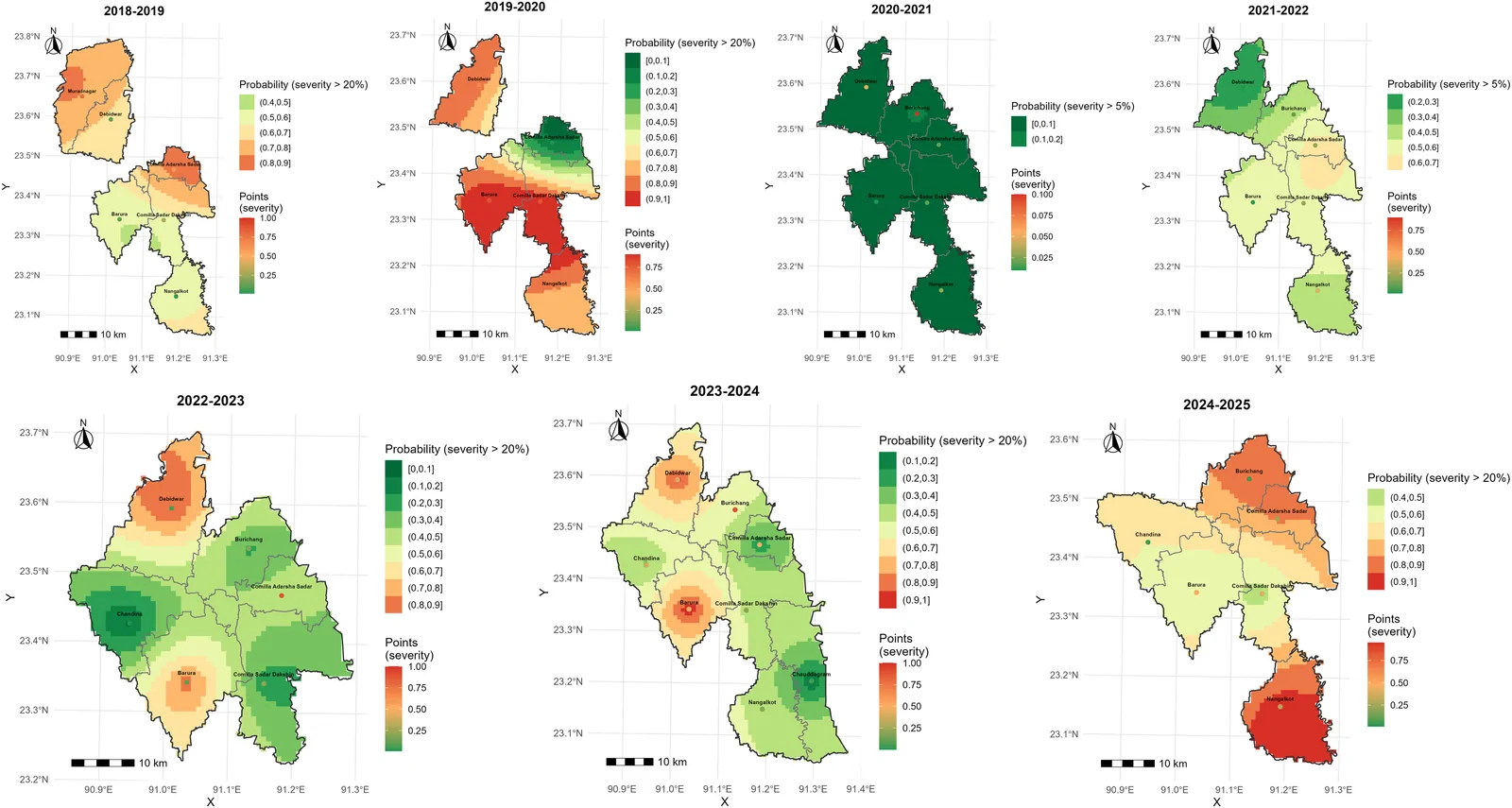
Indicator kriging probability maps of Neck Blast Disease during Boro seasons (2018–19 to 2024–25) in Cumilla. A > 20% severity threshold was used except in 2021–22, when >5% was applied due to low disease severity; therefore, 2021–22 is not directly comparable with other years. Green to red indicates lower to higher probability. Maps were generated using R (version 4.0.3).

Fig 12 uses the indicator kriging approach to examine the spatial probability of Neck Blast Disease severity above 20% across the study Upazilas during the Boro seasons from 2018 to 2025 except 2021 and 2022 where above 5% has been consider for lower prevalence of disease. In 2018–2019, most areas of northern part show the high risk (70%–90%) while other region shows relatively lower risk (40%−60%) than these. In 2019–2020, disease probability intensified sharply, with extremely high risk (>90%) concentrated in central Cumilla Sadar Dakshin and southern Nangalkot and Debidwar and Barura while north part of Cumilla Adarsha Sadar faced less than 20% risk of disease. In 2020–2021, elevated risk persisted in Debidwar, Barura, and some part of central of the district (60%–80%), whereas other regions remained moderate (20%–40%). In 2021–22, overall disease severity was very low; therefore, this season was mapped using a > 5% severity threshold because of the very low overall disease severity and is therefore not directly comparable with years using the > 20% threshold. In 2022–2023, high probability zones (60%–80%) reappeared in Debidwar and Barura, while surrounding Upazilas experienced moderate levels. In 2023–2024, most areas showed relatively lower probability (<40%), except localized hotspots in Debidwar and Barura (70%–90%). The 2024–2025 season again showed strong resurgence, with Burichang, Cumilla Adarsha Sadar, and southern Nangalkot emerging as high-risk zones (>80%), indicating a return of severe blast pressure in these locations.

## Discussion

The spatio-temporal analysis of Neck Blast Disease (NBD) severity revealed a highly heterogeneous distribution across the study area. It underscores the necessity of location-specific, adaptive management strategies. The application of geostatistical techniques such as Local Indicators of Spatial Association (LISA) and Kriging effectively identified persistent disease hotspots and quantified the probability of high-severity outbreaks.

### Spatial and temporal dynamics of NBD vulnerability

The analysis demonstrated distinct vulnerability areas between the T. Aman and Boro rice seasons. In the Aman season (2017–2024), Nangalkot was consistently identified as the most vulnerable Upazila, characterized by the highest mean severity (approximately 45%–60%) and explicated maximum yield loss. Neck blast is the most devastating disease, capable of inflicting catastrophic yield losses ranging from 70% to 80% [34]. Kriging models consistently assigned Nangalkot a greater than 70% risk probability, increasing in a 90% chance of infection in the final year of study. This persistent vulnerability may reflect unmeasured local factors, such as favorable micro-environmental conditions, susceptible rice varieties, cropping practices, or disease-management behavior; however, these factors were not directly measured in the present study [19,35,36]. Conversely, Chandina exhibited low mean severity (0–15%), serving as a control area. The emergence of High–High clusters in Debidwar and Cumilla Adarsha Sadar using LISA indicates significant localized, self-reinforcing disease aggregation points that demand targeted, intensive control measures.

The Boro season (2018–2025) presented a more dynamic epidemiological landscape. While Muradnagar was identified as the overall most vulnerable (45%−60%) Upazila, the spatial distribution of risk zones shifted temporally. High-risk areas, indicated by High–High clusters, moved between Muradnagar, Nangalkot, Debidwar, and Burichang across different years. This spatial non-stationarity indicates that Boro-season Neck Blast risk varied considerably across years and locations [37]. The recent resurgence of high probability zones (>80%) in Burichang, Cumilla Adarsha Sadar, and Nangalkot (2024–2025) warrants immediate risk mitigation efforts in the short term.

To the best of our knowledge, there was no previous study which was done in spatial explicit mode to identify hotspot of Neck Blast Disease in any season in Cumilla District. The results do agree with global research on rice blast. Rice blast is one of the most devastating diseases of rice globally, and it is a major threat to the world food security, according to Asibi et al. [19]. Kihoro et al. showed that rice blast has socioeconomic impacts on the farmers’ livelihood in the Greater Mwea region in Kenya [22]. Similarly, Simkhada and Thapa reported that the weather factors that favor the blast outbreaks are cloudy weather, high relative humidity, low night temperature and long dew duration [34]. A spatial epidemiology study by Amoghavarsha et al. classified the areas of Karnataka, India, as being at a high risk of rice blast in various rice ecosystems and showed how spatial analysis tools like autocorrelation analysis and interpolation methods can be used to detect disease-prone areas [38]. The current results are consistent with the previous studies indicating the complex spatial variability in Neck Blast risk and the need for spatial monitoring and management to mitigate the disease impact.

### Influence of climatic trends

Observed climatic trends between 2017 and 2025 provide context for the observed disease patterns. Both Aman and Boro seasons exhibited an upward trend in both maximum and minimum temperatures. Together with the declining rainfall trend in Aman, these climatic shifts were associated with changes in NBD severity and may have contributed to conditions favorable for disease development.

A notable observation was the lowest NBD severity period during the Boro seasons of 2020–21 and 2021–22. This period coincides with a decline in Relative Humidity (RH) following a peak in 2019–2020. Given that high RH and temperature are critical factors for blast infection, the period of lower Boro RH coincided with reduced NBD severity, suggesting a possible association between humidity conditions and disease development. In a study of environmental factor for rice blast disease, Simkhada K. et al. (2022) demonstrated that the most conducive conditions for disease outbreaks are cloudy weather, high relative humidity, low night temperatures, and prolonged dew duration [34]. However, the subsequent rebound in severity confirms the complex, non-linear relationship between climatic variables and disease expression which suggests that other factors, such as varietal shifts or inoculum pressure, may override marginal climatic suppression. These data emphasize the need for robust predictive modeling that integrates weather variables with spatial context.

### Performance of geostatistical modeling

The performance of the geostatistical models validated their utility in high-resolution risk mapping. Indicator Kriging proved particularly effective by mapping the spatial probability of severity exceeding a critical 20% yield-loss threshold, offering a clear, actionable metric for stakeholders. The variability in the best-fit semivariogram models, Spherical dominance in Aman and a shift to Exponential in later Boro years, confirms the distinct spatial dependence characteristics of the disease in each season. This result is coincided with the study of Amoghavarsha C, et al., (2022) which took place in Karantaka [38]. The spherical model, indicative of a continuous, patch-like distribution, was appropriate for the more established Aman crop cycle, while the exponential model suggests faster diminishing spatial correlation, aligning with the more sporadic and climate-sensitive nature of Boro outbreaks. This finding is critical: effective spatial epidemiology requires season- and year-specific model parameterization to achieve accurate risk assessment.

In summary, the study successfully delineated stable and transient high-risk zones for NBD. Nangalkot and Muradnagar are designated as primary management targets. The dynamic nature of the Boro epidemic, in contrast to the more persistent Aman risk, underscores the critical need for an integrated disease management framework that leverages high-resolution, geostatistical risk maps, informed by seasonal climatic forecasting, to deploy fungicides and resistant varieties strategically.

The interpretation of this study’s results is constrained by several limitations. The most significant limitation is the lack of regional or high-resolution climatic data for the study sub-regions, which prevented precise assessment of the localized effects of rainfall, temperature, and humidity on disease prevalence and intensity. Because the analysis was conducted at the Upazila level, finer field-level variability in NBD severity may not have been fully captured. therefore, the risk maps should be interpreted as Upazila-level patterns intended for regional planning and surveillance rather than as predictions for individual fields. The spatial analysis also did not incorporate cultivar-specific information, crop-management practices, or fungicide-use data, as these were not consistently available across Upazilas and years. Another limitation is the small number of spatial units (7 Upazilas in Aman and 10 in Boro), which may reduce the statistical power and stability of Moran’s I, LISA, clustering, semivariogram fitting, and Kriging predictions. Because no multiple-comparison correction was applied to repeated LISA analyses, significant local clusters should be interpreted cautiously as exploratory spatial patterns. Nevertheless, the multi-year dataset provides valuable insights into persistent and changing NBD patterns across seasons and years. Although cross-validation metrics were used to assess model performance, spatial prediction uncertainty was not mapped explicitly; therefore, the interpolated risk surfaces should be interpreted cautiously, particularly in areas with sparse observations. Future studies should incorporate cultivar-specific resistance profiles, crop-management information, fungicide-use data, finer-resolution spatial units, and high-resolution climatic variables to better explain the spatial and temporal variability of NBD hotspots.

## Conclusion

The present study successfully employed indicator kriging to demarcate the spatio-temporal variation of the risk of Neck Blast Disease (NBD) over the study area, with high spatial heterogeneity and extreme inter-annual variation being evidenced. Spatially, the research identified recurring susceptible areas, with particular emphasis on demarcating Nangalkot as a chronic hotspot area where the highest maximum mean NBD severity was recorded during the Aman season. Temporally, Boro season had complex epidemic cycles, namely sudden intensification (e.g., 2019–2020, in which risk probability was over 90% in various central and southern Upazilas) followed by sudden declines (e.g., 2021–2022). The recurring and very localized re-appearance of high-probability areas, particularly in Debidwar and Barura, confirms that NBD risk is not homogeneous. These findings call for the application of highly location-specific and adaptive monitoring and management strategies to counter these high-probability zones in a bid to avert devastating losses in yields.

## Supporting information

S1 File Year-wise list of study Upazilas by rice season. This table presents the Upazilas included in the study by year and season for Aman and Boro rice ecosystems in Cumilla District.
(PDF)

S2 File Mann–Kendall trend analysis for Aman-season weather variables. This table presents Kendall’s tau, p-values, and trend interpretations for maximum temperature, minimum temperature, rainfall, and relative humidity during the Aman season.
(PDF)

S3 File Mann–Kendall trend analysis for Boro-season weather variables. This table presents Kendall’s tau, p-values, and trend interpretations for maximum temperature, minimum temperature, rainfall, and relative humidity during the Boro season.
(PDF)

S1 Fig Moran’s I values for the Aman season. This figure presents year-wise Moran’s I values used to assess spatial autocorrelation of Neck Blast Disease severity during the Aman season.
(TIFF)

S2 Fig Moran’s I p-values for the Aman season. This figure presents the corresponding year-wise p-values for Moran’s I analysis during the Aman season.
(TIFF)

S3 Fig Moran’s I values for the Boro season. This figure presents year-wise Moran’s I values used to assess spatial autocorrelation of Neck Blast Disease severity during the Boro season.
(TIFF)

S4 Fig Moran’s I p-values for the Boro season. This figure presents the corresponding year-wise p-values for Moran’s I analysis during the Boro season.
(TIFF)
