# Supplementary material for: Delineating season-specific hotspots: A spatio-temporal risk assessment of neck blast disease in rainfed and irrigated ecosystems of Cumilla District, Bangladesh
Source: PLoS One. 2026 Sep 21;21(9):e0358537. doi: 10.1371/journal.pone.0358537 (PMC13592595; doi:10.1371/journal.pone.0358537)
Supplement: Supplementary file 1 [file pone.0358537.s001.pdf]

Table 1: Upazila List by Season

Year	Season	Upazillas
2,017	Aman	Adarsha Sadar, Barura, Burichang, Debidwar, Nangalkot, Sadar dakshin
2,018	Aman	Adarsha sadar, Barura, Debidwar, Lalmai, Nangalkot, Sadar Dakshin
2,019	Aman	Adarsha Sadar, Barura, Burichang, Debidwar, Nangalkot, Sadar Dakshin
2,020	Aman	Adarsha sadar, Barura, Burichang, Debidwar, Nangalkot, Sadar Dakshin
2,021	Aman	Adarsha Sadar, Barura, Burichang, Debidwar, Nangalkot, Sadar Dakshin
2,022	Aman	Adarsha sadar, Barura, Debidwar, Nangalkot, Sadar Dakshin
2,023	Aman	Adarsha Sadar, Barura, Burichang, Chandina, Debidwar, Nangalkot, Sadar dakshin
2,024	Aman	Adarsha Sadar, Barura, Burichang, Debidwar, Nangalkot, Sadar Dakshin
2,018	Boro	Adarsha sadar, Barura, Debidwar, Muradnagar, Nangalkot, Sadar Dakshin
2,019	Boro	Adarsha Sadar, Barura, Debidwar, Nangalkot, Sadar Dakshin
2,020	Boro	Adarsha sadar, Barura, Burichang, Debidwar, Laksham, Nangalkot, Sadar Dakshin
2,021	Boro	Adarsha Sadar, Barura, Burichang, Debidwar, Laksham, Nangalkot, Sadar Dakshin
2,022	Boro	Adarsha Sadar, Barura, Burichang, Chandina, Debidwar, Laksham, Lalmai, Sadar Dakshin
2,023	Boro	Adarsha Sadar, Barura, Burichang, Chandina, Chauddagram, Debidwar, Laksham, Nangalkot, Sadar Dakshin
