# Supplementary material for: Delineating season-specific hotspots: A spatio-temporal risk assessment of neck blast disease in rainfed and irrigated ecosystems of Cumilla District, Bangladesh
Source: PLoS One. 2026 Sep 21;21(9):e0358537. doi: 10.1371/journal.pone.0358537 (PMC13592595; doi:10.1371/journal.pone.0358537)
Supplement: Supplementary file 2 [file pone.0358537.s002.pdf]

Table 2: Mann–Kendall trend analysis for the Aman season

Variable	Kendall's tau	p-value	Interpretation
Maximum temperature	0.282	0.041	Significant increasing trend
Minimum temperature	0.197	0.156	Non-significant increasing tendency
Rainfall	−0.088	0.532	Non-significant decreasing tendency
Relative humidity	−0.214	0.123	Non-significant decreasing tendency
