# Supplementary material for: Delineating season-specific hotspots: A spatio-temporal risk assessment of neck blast disease in rainfed and irrigated ecosystems of Cumilla District, Bangladesh
Source: PLoS One. 2026 Sep 21;21(9):e0358537. doi: 10.1371/journal.pone.0358537 (PMC13592595; doi:10.1371/journal.pone.0358537)
Supplement: Supplementary file 3 [file pone.0358537.s003.pdf]

Table 3: Mann–Kendall trend analysis for the Boro season

Boro weather variable	Kendall's tau	p-value	Interpretation
Maximum temperature	0.233	0.224	Non-significant increasing tendency
Minimum temperature	−0.017	0.964	No clear trend
Rainfall	0.109	0.589	Non-significant increasing tendency
Relative humidity	−0.250	0.192	Non-significant decreasing tendency
